# The influence of HARP (The Health Access for Refugees’ Project) on vaccine hesitancy in people seeking asylum and refugees in Northern England

**DOI:** 10.1080/16549716.2025.2457808

**Published:** 2025-02-03

**Authors:** Marie-Clare Balaam, Melanie Haith-Cooper

**Affiliations:** a School of Nursing and Midwifery, University of Central Lancashire (UCLan), Preston, UK; b Faculty of Health Studies, University of Bradford, West Yorkshire, UK

**Keywords:** Asylum seekers and refugees, vaccine hesitancy, COVID-19 vaccine uptake, grassroots interventions, public health, peer support

## Abstract

**Background:**

Evidence suggests that people who are asylum seekers and refugees experience poorer physical and mental health compared to the general UK population and poor outcomes from COVID-19 if unvaccinated. However, this population can experience vaccine hesitancy and other barriers inhibiting their up-take of the COVID-19 vaccine.

**Objectives:**

This study explored the influence of HARP (Health Access for Refugees’ Project) workshops on the intention to have the vaccine in people who are asylum-seekers and refugees.

**Methods:**

A qualitative study including clients (asylum-seekers and refugees), volunteers and HARP staff was undertaken to explore perceptions of HARP workshops and their influence on the barriers to the uptake of the COVID-19 vaccine including vaccine hesitancy. Semi-structured telephone interviews were undertaken with 10 participants, HARP clients (*n* = 1), HARP volunteers (*n* = 6, of whom 4 had been clients) and staff (*n* = 3). Data were thematically analysed.

**Results:**

Intention to have the vaccine was influenced by trusted sources including peers and health professionals. Tailoring evidence-based information to individuals and challenging misinformation were important influencers on vaccine uptake. HARP activity increased the uptake of vaccines in large accommodation centres and hotels. Grassroots-level interventions such as HARP workshops appear to increase intention to take up the COVID-19 vaccine in asylum seeking and refugee communities.

**Conclusion:**

This model could be adopted for health screening such as breast cancer and other vaccinations within asylum seeking and refugee communities.

## Paper Context

- **Main findings**: Intention to have the vaccine and vaccine uptake was influenced by trusted sources, such as peers and health professionals, providing tailored and evidence-based information which challenged misinformation and gave individuals increased confidence in vaccination.
- **Added knowledge**: Grassroots-level interventions which work within the community appear to increase intention to take up the COVID-19 vaccine in asylum seeking and refugee communities.
- **Global health impact for policy and action**: This model has implications for the promotion and uptake for various public health initiatives including health screening and other vaccinations within asylum seeking and refugee communities.

## Background

People seeking asylum and refugees (ASR) have disproportionately poor physical and mental health compared to the general UK population [1] including poor outcomes from COVID-19 if unvaccinated [2,3]. A UK survey found that vaccine hesitancy is common in people from ASR backgrounds, this has been attributed to misinformation on social media [4–7], concerns about the effectiveness, composition and possible side-effects of the vaccine [5,8,9] and perceived conflicts with religious and cultural beliefs [10,11]. ASR can also lack trust in UK authorities and may fear being charged for vaccines or their data being shared with the Home Office [7–9]. To address vaccine hesitancy in this population, accurate, accessible and culturally sensitive information needs to be provided, from a trusted source such as a member of the local community [5], in a number of languages [7,12] and in a convenient location [5].

The Health Access for Refugees Project (HARP) is delivered across five areas in Northern England by the Refugee Council. The aim is to improve the physical and mental health of ASR and to help reduce health inequalities. During the COVID-19 pandemic, HARP was ideally placed to deliver an intervention to promote vaccine uptake in ASR clients through tailored workshops delivered by trained peer volunteers and HARP staff. Initially, local GPs and public health professionals were invited to the workshops to provide accurate information about the vaccination, challenge misinformation and facilitate HARP staff and volunteers’ confidence for future workshop delivery. Information from the workshops was translated into different languages and disseminated to the wider refugee community through a WhatsApp group. Workshops were also integrated into existing HARP interventions such as health conversation clubs, health briefings for new arrivals in the country and information was then re-enforced through a one-to-one telephone peer befriending programme (Table 1).

**Table 1.** HARP activities.

Initial accommodation health briefings
One-to-one telephone befriending service
English for Health
Weekly groups: Art therapy, health conversation club, healthy cooking, allotment group
COVID-19 vaccine workshops
One to one health advocacy and support
Volunteer training
Awareness raising sessions – public & professionals

Peer volunteers are provided with a range of training opportunities and are integrated throughout all aspects of the project, providing one-to-one support to clients as well as involvement in group settings. The role of peers is a crucial and innovative part of HARP and is explored in depth elsewhere [13,14]. As part of a wider HARP evaluation [14] we undertook a qualitative study to address the following research questions:

1. What is the impact of the HARP workshops on intention to uptake the COVID-19 vaccine in ASR?
2. How has HARP facilitated volunteers and staff to address wider institutional and system-level barriers to ASR take up the COVID-19 vaccine?

## Methods

A qualitative design was used to explore in-depth the perceptions of participants. Ethics approval was obtained from the University of Bradford (Reference EC26224). As part of the ethics procedure, it was agreed that informed consent for participation in the study would be obtained verbally. This was done to reduce participants’ concerns about signing documentation and is a method which has been used in other studies with ASR [15]. Audio recorded semi-structured telephone interviews were chosen as the most appropriate method for this research. This approach allowed the researchers to address ongoing issues related to COVID-19 restrictions, concerns about face-to-face research and the limited budget of the study. This approach was one which was acceptable to potential participants who are used to HARP contacting them in this way and enabled us to potentially reach participants who may have left the area. Interviews were undertaken by MCB with 10 participants who were HARP clients (*n* = 1), volunteers (*n* = 6, of whom 4 had previously been clients) and staff (*n* = 3). The interview schedule was co-produced with HARP staff and volunteers who were asylum seekers and refugees. The questions explored the impact of HARP interventions on intention to take up the COVID-19 vaccine, volunteers’ experience of facilitating the COVID-19 workshops and how staff addressed institutional and systems barriers to increase vaccine uptake (see Table 2).

**Table 2.** Interview schedule.

**Clients** (1) Before the HARP workshop, what was your opinion of the COVID vaccine? Were you planning on having the vaccine? Why? (2) After the HARP workshop, what was your opinion of the COVID vaccine? Did it change your opinion on whether to have the vaccine? Why? (2) What do you remember most about the workshop? Which aspect (if any) affected your decision about the COVID vaccine? (4) Have you had your COVID vaccine? If not, why not? **Additional questions for volunteers** (1) What has been your role in relation to the COVID vaccine? What training/preparation did you have for this role? Did you feel well prepared? (2) Do you think you influenced people’s decision about whether to have the COVID vaccine or not? How do you think you did this? (3) What do you think are the main reasons why people decide not to have the COVID vaccine or decide to have the COVID vaccine? (4) Can you think of any other ways we could support people who are asylum-seekers and refugees to have the COVID vaccine? **Questions for staff.** Working as a member of staff for HARP, do you think you have had the opportunity to make changes which has helped clients have the COVID vaccine? In what ways? e.g., working with other organisations, instigating policy or practice changes, expanding current services

Participants were purposively selected using sample stratification [16] to ensure that there were multiple perspectives represented and to acknowledge the heterogeneity of experiences of refugees and those seeking asylum. HARP staff supported recruitment by contacting potential participants by telephone. They used an information sheet to explain the purpose of the study and the consent, and data sharing procedures. They then referred potential participants, who had agreed for their contact details to be shared, to the research team who then contacted the individuals directly. Interpreters were offered but none were required. Confidentiality was ensured in all aspects of data transfer and all electronic files were stored on a password protected University drive and subsequently deleted in accordance with data protection procedures.

Data were anonymised and transcribed verbatim by a professional organisation. Data were then subject to inductive thematic analysis [17]. Following familiarisation with the data, which was achieved through reading and re-reading the transcripts, MCB, undertook line by line coding on several transcripts by searching for patterns in the data. From this an initial coding scheme was developed, this was then reviewed by MC, following this the remaining transcripts were coded. These codes were then grouped into initial themes which were reviewed by MC and discussed by both authors to reduce researcher bias. Final themes were agreed by both authors, see Table 3.

**Table 3.** Themes.

Codes	Working themes	final themes
Social media as source of knowledge	Understanding the challenges to accessing information about COVID & vaccinations	Filling the knowledge gap
Family as sources of knowledge		
Medical profession as sources of knowledge		
Lack of knowledge		
Language barriers		
Beliefs about COVID	The need to acknowledge and address misinformation	Countering the impact of false information
Who to trust		
Where to go for information		
False and misinformation		
Challenging misinformation		
People like me	Peers as trusted individuals	The influence of peers
Shared language, culture and experiences		
Peers as role models		
Trust		
Share vaccination experience		
No data to access online system to book appointments for vaccine	Practical barriers to accessing vaccinations	Going beyond the workshops
Lack of English language		
Difficulty accessing vaccination centres		
Providing data to support access to internet	Solutions to overcome these barriers	
Provision of language support online & in person		
Working with local authorities		
Working with accommodation centres		
Timing of vaccinations		
Location of vaccination centres		

## Results

All volunteer and client participants were seeking asylum or refugees, they came from seven different countries in Africa, South America, Europe, and the Middle East. They were all aged between 30 and 49, had lived in the UK between 1 and 14 years and over half (*n* = 7) identified as women (see Table 4). All staff were female and UK citizens, no other demographic data was collected from staff. Four key themes were developed from the data. Notations for the quotations are, S = staff member, V = HARP volunteer, C/V = previous client now acting as volunteer and C = client.

**Table 4.** Volunteer and client participant demographics.

	Role	Gender	Time in UK	Home region
1_V	Volunteer	F	12 years	Africa
2_V	Volunteer	M	14 years	Middle East
1_C/V	Client/volunteer	M	15 months	Middle East
1_C	Client	F	2 years	Europe
2_C/V	Client/volunteer	M	2 years	El Salvador
3_C/V	Client/Volunteer	F	22 months	Iraq
4_C/V	Client/Volunteer	F	3 years	Albania

## Filling the knowledge gap

Before the HARP workshops, many participants lacked knowledge about COVID-19 and were uncertain about whether to have the vaccine. Staff explained how ASR faced difficulties in accessing reliable information through social media or the internet, due to language barriers or a lack of credit/data on their mobile phones:

A lot of people just didn’t know that much about it [vaccine] because they haven’t had the digital inclusion to really be up-to-date on the right stuff. (1_S)

Clients and volunteers also discussed having limited social networks and not knowing who to trust when receiving information on whether to have the vaccine:

We didn’t know any friends here, we are alone, that’s to say only my family and we didn’t know who to ask, what to do. (1_C)

Following the HARP workshops, participants reporting understanding the benefits of the vaccine. Including health professionals in delivery was an important factor in facilitating uptake of the vaccine:

… we had discussion with GP, a doctor, and she explained everything to us that the vaccine was considered to be safe … and that’s why after that we changed our mind and me and my husband, we are fully vaccinated. (1_C)

Providing tailored information to address clients’ specific health needs directly resulted in them having the vaccine:

The doctor explained to me, she said, no, no problem, even if you have allergy the Penicillin, this is something different in the vaccine and I took the vaccine… . (3_C/V)

The HARP workshops built on the information participants had previously received from the NHS, which increased their confidence to take up the vaccine:

My husband received, first of all, an SMS on the phone and after that a letter and … the diabetic nurse called him and told him to have it, to come and have the vaccine. But she told him 2 weeks before or, I don’t remember, 3 weeks before the date. During all this time he didn’t know what to do. When we had this session the GP of the HARP session, after that he decided, and he went. (1_C)

Several participants discussed the significance of having the time and space within the workshops to ask their questions and raise issues that concerned them:

I can ask my questions and I got good answers. I’m learning more about the vaccine, about the effects, about the good things for me if I had the vaccine, and actually I had the two doses from AstraZeneca. (2_C/V)

## Countering the impact of false information

Before the HARP intervention, many participants received misinformation about the vaccine. This came from friends and communities and for those with internet access, through social media, one client explained how:

We weren’t very sure and a lot of maybe fake information was on the internet and some people said that if you have the vaccine maybe you’ll die or maybe you’ll have a blood clot or your genes will be changed. (1_C)

Staff reported that the workshop directly challenged the misinformation participants had heard:

The GPs were so fantastic, they really went into depth with them you know, like, ‘why do you think that’? ‘Where did you hear that news from’? Like, ‘this is the science behind it, this is what we know’, … so like she really went into, like explored that fear with them and, ‘why do you think this’? And unpicked it. (2_S)

They also noted that false information previously received by clients was challenged by directing them to more trustworthy sources of information:

We spent a lot of time, you know, talking to them about … like please use official sources, trusted sources … that are reliable … do not trust, you know, [what] people [say] because you don’t know where these things have [come from] …We tell them to use the NHS or this other great one that the GP told them about. (3_S)

Misinformation also originated from the UK asylum system which HARP staff successfully challenged:

… a horrendous letter from the Home Office … I can’t remember the exact wording but it’s something like, you know ‘if you don’t take the vaccine this will not help your case and you could be deported’, something really bad like that … I mean shocking … I took it to our advocacy team in the Refugee Council as well and they complained to the Home Office at a higher level … and we got the letter withdrawn (1_S)

## The influence of peers

Language was a major barrier to receiving information about the COVID-19 vaccine and peers interpreting information in workshops had a powerful effect on intention to have vaccines. One member of staff noted that:

I believe it’s hearing it in their own language from someone who looks like them, speaks like them… . I could say it, you know, until I’m blue in the face but then you get one of the Sudanese volunteers to go up and say, you know, what’s the problem, I’ve got mine and say it to them in their own language. I think it’s priceless. (3_S)

Peers sharing a cultural and religious background helped address specific concerns:

So hearing from me it’s more easier for them to hear [than] from a British one… . I got lots of questions about the culture itself, like religious and even some of them was believing in it had pork or gelatine … (2_V)

Peers were also a trusted source of information as they had a shared understanding of clients’ life experiences:

They will not discuss it with a white person, they are really worried about their claim and, you know, some of them think if I argue about the vaccine, they will not accept my claim … so if they hear the answer from one like me it will be usually different very, because I am one of them, you know. Like I went through what you went through and I know what I’m talking about. (2_V)

Peers discussing their own vaccination experiences engendered trust, having a positive impact on vaccine uptake:

They say no, we will not have it. I’m so scared, I’m so afraid, then after, but I was the first person I had it. I was the first person and they wow, they say wow, she’s pregnant, she’s having a COVID. [vaccination] (4_C/V)

## Going beyond the workshops

HARP staff and peers helped clients to overcome wider barriers to vaccine uptake. This included practical barriers to accessing vaccine clinics. One peer who had previously been a client explained:

I am living in a sharing accommodation, and I have one housemate, she’s not speaking in English, and I booked for her the appointment to take the vaccine. (3_C/V)

HARP also addressed barriers to attending the online HARP workshops, providing mobile data, and ensuring language support for clients at the workshops:

We had 60 participants, divided them into different language groups, so we had I’d say about 10, around 10 on each session and yes, ran 6 sessions in different language groups. (2_S)

Face-to-face interventions were also delivered in hotels and accommodation centres where ASR were living. This was followed by pop-up vaccination clinics soon after the workshops facilitated by HARP liaising with the local authority. One member of staff noted what they saw as the efficacy of the intervention in increasing uptake of the vaccine in one of the accommodation centres:

There’s over 100 men … housed there so we did in-person ones there … we got just under 60 people and yes again very engaged, asking lots of questions, and the impact, we got kind of a direct impact from that one because all the men were vaccinated at the hotel so it was very easy to follow-up who was and who was not vaccinated … because we planned it so that … we did the sessions the week before, so it was fresh in their memory, and he [accommodation centre staff] said that over 80% of the men were vaccinated which he said was, he thinks was a real direct result of the sessions that we did, he was very happy, so that’s a massive impact. (2_S)

## Discussion

This study aimed to explore the influence of co-developed, and peer facilitated workshops on COVID-19 vaccine hesitancy in people who are ASR, from the perspectives of staff, volunteers, and clients most of whom had gone on to become volunteers. The workshops had a significant impact on intention to take up the vaccine. Involving trusted health professionals was effective in providing tailored information and answering questions to meet individual clients’ needs and to overcome misinformation about the vaccine. Peers had a powerful influence due to shared language, culture, religion and lived experience which built trust. HARP’s role in ensuring accessibility of workshops and vaccination clinics led to an increased uptake of the COVID-19 vaccine.

Barriers to health service uptake in ASR exist at individual, institutional and systems levels [18]. In relation to the COVID-19 vaccine, we found individual barriers around a lack of understanding of the need for vaccination. This supports previous work which found vaccination information culturally and linguistically inappropriate for ASR and that ASR expressed concerns over the need for vaccination and safety and efficacy [5,8–10,19]. A recent systematic review of public health interventions to support refugee access to health services, identified peer support, translation services, health education, accessible interventions, and multidisciplinary approaches as being vital [18]. The HARP COVID-19 vaccine workshops included these key aspects through involving peers in intervention delivery, providing information in ASR languages, while also ensuring a multidisciplinary approach including doctors and other trusted health professionals in intervention delivery.

Research suggests that to address vaccine hesitancy in this population information needs to be from a trusted source and trust was an important issue in addressing individual-level barriers at the workshops. This related to trusting the information provided by health professionals, but also related to the role of peer in the intervention delivery. Research suggests that peers can function as trusted sources when delivering public health information, helping to increase the intention to change health behaviour [20]. Using peers, individuals with similar lived experience, in this case of forced migration, or shared cultural or religious background, can lead to a sense of connection, belonging and community, building trust and a more authentic and equal relationship within support interventions [21–24]. The cultural competence of health professionals and culturally responsive practices are an important aspect of effective primary care for ASR [25,26] and these factors are also important facilitators of vaccine uptake [27]. A trusting relationship, clear communication and cultural understanding are all elements of cultural competence and involving peers provides a way of addressing these issues and supporting culturally competent public health messaging.

As well as addressing some individual-level barriers, HARP had an impact upon some of the institutional and systems-level barriers which have been identified as having a negative impact on vaccine uptake, including the use of hostel accommodation and other aspects of immigration housing policy and practice [28]. They did this through working closely with other local and national agencies and sharing knowledge and good practice to bring about policy change. Their work also facilitated a more effective co-ordination of services, an element identified by Crawshaw et al. [27], as important for vaccine uptake. Additionally, staff felt that HARP’s initiative to facilitate the delivery of vaccines soon after the workshops, in accommodation centres and hotels where the clients were housed, directly increased vaccine uptake. This resonates with work suggesting inaccessible locations create a barrier to vaccine up-take [5,9,19] whereas primary care facilities located close to the target population achieve the highest levels of access [18]. The importance of the community-based nature of interventions like HARP resonates with research which suggests that working with communities and providing vaccinations in community-based or alternative non-health care locations supports uptake of the vaccine [5,12,25,27].

## Limitations of this study

The numbers in the study are small, although they do represent participants with a range of key demographics; however we acknowledge that there is a lack of diversity in the age of the participants, which may have limited the perspectives represented in the study. There were challenges recruiting clients, partly due to the COVID-19 pandemic, which meant that there were still some restrictions on face-to-face research and that some individuals were reticent to meet in person. Additionally, some previous clients had been dispersed to new areas and had not left a contact phone number with HARP or the Refugee Council. This means that the paper presents a limited perspective from clients who did not become volunteers and more research needs to be done with clients who did not go on to volunteer, as they may have a less positive view of the service. However, the study presents new findings which add to existing knowledge by exploring the role of peers as a key aspect of an intervention and provides a case study of a successful approach to supporting COVID-19 vaccination uptake which could be replicated at larger scale.

## Conclusion

This study demonstrates the way in which a grassroots-level intervention increased COVID-19 vaccine intention and uptake amongst ASR. The model of delivering workshops where tailored information is imparted by trusted health professionals and peers, followed by a pop-up vaccination centre in large hotels and other accommodation centres could be applied to areas where other marginalised communities meet such as hostels for the homeless. The model could be used to facilitate uptake of childhood vaccines, tuberculosis (TB) and human papilloma Virus (HPV) and applied to NHS health screening programmes such as cervical, breast and bowel screening. Integrating peer volunteers into this model was crucial to success of the intervention and could be adopted in other contexts co-designing and then implementing the intervention. Due to limited time and resources, we were unable to identify the impact of the HARP intervention on wider vaccine uptake in ASR. Further research is needed to explore the impact on uptake and continued take up of other vaccinations in the future.
